# Arthritis Deformans

**Published:** 1910-06

**Authors:** 


**Arthritis Deformans.** By R. Llewellyn Jones, M.B. Lond. Pp. xiv, 365. Bristol : John Wright & Sons Ltd. 1909.

Dr. Jones has in this volume, with the sub-title, comprising "Rheumatoid Arthritis, Osteo-arthritis, and Spondylitis Deformans," given a most excellent epitome of present-day knowledge of the diseases of the rheumatoid class. Agreeing with most modern workers, he makes a sharp distinction between the two diseases, rheumatoid arthritis and osteo-arthritis, nor does he believe that the former may merge into the latter. Another sign of our increased knowledge of these diseases is the gradual emergence of rheumatoid arthritis as a disease distinct from various septic and tubercular joint infections ; whilst the condition known as Still's disease is here shown to be not a separate entity, but to be made up partly of atypical cases of tuberculosis and very largely of cases of rheumatoid arthritis modified by its occurrence in young children. Three-fifths of Dr. Jones's work is taken up with the consideration of rheumatoid arthritis. The author discards the modern theory of a bacterial origin, and very ably and convincingly supports the view that the condition is a spinal toxæmia, or toxic tropho-neurosis of gastro-intestinal origin. The chapters on pathogeny and on premonitory symptoms are of the best in the book, and will well repay study, although possibly the author's views will later on need revision when a further examination of the disease has been made in the light of the opsonic index and vaccine therapy. The author's well-known views on the subject of the nervous and vaso-motor symptoms of rheumatoid arthritis will be found here fully expounded, and will be read by all with interest and profit. With regard to osteo-arthritis and its causation, we find, after eliminating cases of proliferative arthritis due to tubercle, trauma and sepsis, that the two theories which hold the field to-day are, firstly, that the condition is part of a general metabolic change accompanying widespread arterio-sclerosis, or, secondly, that it is due to the withdrawal or perversion of one or more of the internal secretions, such as that of the thyroid or ovary. The writer supports the internal secretion theory, and it is one which is certainly growing in favour with the medical profession. The chapters devoted to treatment are full of good, sound, practical advice, and are the more valuable as being written by one who is thoroughly conversant with modern balneo-therapeutic measures, Dr. Llewellyn Jones is to be congratulated upon the very excellent illustrations which are so profusely provided, and upon the exhaustive bibliography, which adds so much to the value of his book, and which will help to make it the standard work on rheumatoid conditions for many years to come.

